# Exposure to particulate matters and risk of diabetes-related mortality: a systematic review and meta-analysis

**DOI:** 10.1265/ehpm.25-00424

**Published:** 2026-03-07

**Authors:** Weifang Yang, Jing Li

**Affiliations:** 1 Department of Endocrinology, Qingdao Municipal Hospital, Qingdao, 266071, China; 2 Department of Cadre Health Care, Qingdao Municipal Hospital, Qingdao, 266071, China

**Keywords:** Particulate matter, PM_2.5_, PM_10_, Diabetes-related mortality, Meta-analysis

## Abstract

**Background:**

Epidemiological evidence increasingly implicates ambient particulate matter (PM) as a contributor to diabetes progression and premature mortality; however, the strength and consistency of these associations remain uncertain. This systematic review and meta-analysis quantified the relationship between exposure to major PM fractions—particularly PM_2.5_ and PM_10_—and the risk of diabetes-related mortality in adult populations.

**Methods:**

Following PRISMA and MOOSE guidelines, five databases were searched through September 30, 2025. Eligible observational studies assessed long- or short-term exposure to ambient PM and reported quantitative estimates for diabetes-associated deaths. Effect sizes were pooled using random-effects models and expressed as relative risk (RR) per 10 µg/m^3^ increment in PM concentration. Subgroup and meta-regression analyses explored sources of heterogeneity.

**Results:**

Thirty-six studies (41 datasets) were included. Pooled analysis showed a significant positive association between PM_2.5_ exposure and diabetes-related mortality (RR = 1.123; 95%CI: 1.099–1.147; I^2^ = 95.3%). The effect was stronger for long-term exposure (RR = 1.296; 95%CI: 1.197–1.395) and prospective cohorts (RR = 1.327; 95%CI: 1.189–1.466). PM_10_ was also associated with increased risk, though with smaller magnitude (RR = 1.021; 95%CI: 1.007–1.035; I^2^ = 81.7%). Meta-regression confirmed exposure duration as a significant modifier (p = 0.014).

**Conclusions:**

Both fine (PM_2.5_) and coarse (PM_10_) particulate matter are significantly associated with increased diabetes-related mortality, with the strongest and most consistent effects observed for chronic PM_2.5_ exposure. These findings highlight the metabolic health burden of air pollution and underscore the importance of stringent air-quality standards to reduce premature diabetes deaths globally.

**Supplementary information:**

The online version contains supplementary material available at https://doi.org/10.1265/ehpm.25-00424.

## Introduction

Diabetes mellitus (DM) has become one of the most pressing global health challenges of the 21st century. Recent estimates indicate that more than 529 million adults worldwide are living with diabetes, with projections suggesting a rise to over 1.31 billion by 2050 [1] (Sun et al., 2023; IDF, 2024). The International Diabetes Federation further estimates that diabetes was responsible for some 3.4 million deaths in 2024, equivalent to one death every nine seconds [2]. Nearly half of all diabetes-related deaths occur before the age of 70, reflecting the enormous impact of premature mortality [3]. Beyond direct deaths, diabetes substantially contributes to cardiovascular disease, renal failure, neuropathy, and other life-limiting complications, leading to significant health-care expenditures and productivity loss [4]. The disease burden is driven by a complex web of factors, including obesity, sedentary lifestyle, unhealthy diet, smoking, and genetic predisposition [5, 6]. In recent years, however, growing attention has turned to environmental exposures—particularly air pollution—as potential modifiers of diabetes risk and outcomes [7, 8].

Air pollution is now recognized as one of the leading global environmental threats, responsible for an estimated 6–7 million premature deaths annually [9]. Among its components, particulate matter (PM) plays a central role in driving adverse health outcomes [10]. PM is generally categorized by aerodynamic diameter, with PM_2.5_ (particles ≤2.5 µm) capable of penetrating deep into the lungs and entering the bloodstream, and PM_10_ representing coarser particles [11]. Major sources include vehicle emissions, industrial processes, fossil fuel combustion, and biomass burning [12]. Chronic exposure to PM has been linked to cardiovascular and respiratory diseases, and increasing evidence suggests it also contributes to metabolic disorders such as diabetes [11]. Biologically, PM may promote diabetes-related mortality through several interrelated mechanisms: it induces systemic inflammation, oxidative stress, and endothelial dysfunction, all of which can aggravate insulin resistance, impair pancreatic β-cell function, and accelerate vascular complications [13, 14].

Epidemiological research over the past decade has increasingly explored the association between ambient PM exposure and diabetes incidence, glycemic control, and mortality [13, 15]. Several cohort studies have shown that long- or short-term exposure to PM_2.5_ is associated with higher fasting glucose levels, increased HbA_1_c, and elevated diabetes prevalence [15–17]. By 2021, the global disability-adjusted life years (DALYs) linked to type 2 DM from exposure to PM_2.5_ had risen markedly to approximately 12.9 million, representing nearly a threefold increase compared with 4.6 million DALYs in 1990 [18]. However, evidence specifically linking PM exposure to diabetes-related mortality remains limited and inconsistent. Earlier meta-analysis was constrained by a small number of studies and methodological heterogeneity [13], while newer research has reported variable effect estimates across different populations and exposure metrics.

Given these uncertainties and the growing relevance of environmental determinants in chronic disease outcomes, an updated and comprehensive synthesis is needed. The present systematic review and meta-analysis aims to quantitatively assess the association between PMs exposure and diabetes-related mortality.

## Methods

### Study design and search strategy

The methodology of this study followed the Preferred Reporting Items for Systematic Reviews and Meta-Analyses (PRISMA 2020) and Meta-analysis of Observational Studies in Epidemiology (MOOSE) guidelines [19, 20]. The review protocol was prospectively registered in the International Prospective Register of Systematic Reviews (PROSPERO) [Registration ID: CRD420251167309].

A comprehensive literature search was performed across the following electronic databases: PubMed/MEDLINE, Web of Science, Embase, Science Direct, and Scopus from inception to September 30, 2025. To identify grey literature, we also searched ProQuest Dissertations, WHO Global Index Medicus, and reference lists of relevant reviews. The reference lists of all eligible studies and related reviews were hand-searched to identify additional publications. The search strategy combined controlled vocabulary (MeSH/Emtree terms) and free-text words related to air pollution, particulate matter, diabetes, and mortality. Search terms were adapted for each database (Table S1).

### Eligibility criteria

Eligibility criteria were defined according to the PECO framework—Population, Exposure, Comparator, and Outcome—to ensure the inclusion of relevant and methodologically sound evidence. The population (P) comprised adults aged 18 years or older diagnosed with diabetes mellitus (type 1 or type 2), or general population cohorts in which diabetes-related deaths were specifically identified. The exposure (E) of interest was ambient PM, including fine particles with aerodynamic diameters ≤2.5 µm (PM_2.5_), inhalable coarse particles ≤10 µm (PM_10_), or total suspended particles (TSP). Eligible studies were required to report quantitative exposure assessments obtained from fixed-site monitoring networks, satellite-based estimations, or validated atmospheric dispersion models, which serve as proxies for population-level exposure, with exposure expressed in µg/m^3^ or comparable units. The comparator (C) consisted of lower exposure categories, reference concentrations, or populations with minimal or background levels of PM exposure. The primary outcome (O) was diabetes-associated mortality, defined as deaths in which DM was listed as an underlying or contributing cause of death, identified using International Classification of Diseases (ICD-9: 250; ICD-10: E10–E14) or equivalent diagnostic coding systems.

Eligible studies were required to adopt an observational analytical design, including prospective or retrospective cohort studies, nested case-control studies, or population-based ecological studies that provided a quantitative estimate of association—such as relative risk (RR), hazard ratio (HR), or odds ratio (OR)—with corresponding 95% confidence intervals. Ecological studies were included to capture a broader range of geographical and temporal contexts, with the understanding that their results require careful interpretation due to the limitations of the design. To ensure methodological robustness, only original researches published in peer-reviewed journals without any language restriction were considered. Studies were included if they examined long- or short-term exposure to PM in relation to diabetes mortality, adjusted for major confounding factors such as age, sex, and comorbid conditions. Studies were excluded if they met any of the following criteria: (1) did not provide quantitative effect estimates specific to diabetes-related mortality; (2) investigated indoor air pollution, occupational exposure, or mixtures without isolating the effects of PM; (3) were reviews, conference abstracts, commentaries, editorials, or case reports; or (4) involved overlapping populations with other eligible studies, in which case the most comprehensive or recent dataset was retained.

### Data extraction

A standardized data extraction form was developed in Microsoft Excel. For each study, we collected the following information: (1) Bibliographic details: first author, publication year, country, and study design. (2) Population characteristics: sample size, age, sex distribution, and diabetes type (if specified). (3) Exposure assessment: pollutant type (PM_2.5_, PM_10_, etc.), exposure period (short-term vs. long-term), measurement technique, and mean or median concentration levels. For this review, ‘short-term exposure’ refers to studies assessing associations with daily or sub-daily variations in PM concentration, while ‘long-term exposure’ refers to studies assessing associations with average PM concentrations over periods of one year or more. (4) Outcome data: number of diabetes-related deaths, follow-up duration, and effect estimates with 95% Cis. (5) Adjustment variables: confounders included in multivariable models (e.g., age, sex, BMI, smoking, socioeconomic status, comorbidities) (Table S2). When multiple models were reported, estimates adjusted for the most confounders were extracted. For studies with multiple cohorts and same population, we used data from last cohort. When data were missing, we contacted corresponding authors for clarification.

### Quality assessment

The risk of bias (RoB) and methodological quality of the included studies were assessed independently by two reviewers using design-specific tools. For cohort studies, we employed the Newcastle-Ottawa Scale (NOS) [21], which judged studies based on three domains: the selection of the study groups, the comparability of the groups, and the ascertainment of either the exposure or outcome of interest. For case-crossover and time-series studies, we used the Risk Of Bias In Non-randomized Studies - of Interventions (ROBINS-I) tool [22]. This tool was applied with a focus on domain-specific concerns such as bias due to confounding—particularly from time-varying factors like seasonality and temperature—and bias in the selection of the reported result related to model specification [23, 24]. For ecological studies, we applied the Risk Of Bias in Ecological Studies (ECO) tool, an extension of ROBINS-I developed to address specific methodological challenges, such as ecological fallacy, inherent in this design [25]. Any discrepancies between the reviewers’ assessments were resolved through discussion or by consultation with a third reviewer. Based on these tools we categorized studies as low, moderate and high qualities.

### Data synthesis and statistical analysis

To ensure consistency for meta-analysis, all effect estimates for PM were harmonized to represent the risk ratio (RR) associated with a 10 µg/m^3^ increment. The included studies reported associations using different metrics, including hazard ratios (HR), odds ratios (OR), and percentage changes in risk per study-specific Interquartile Range (IQR) (Table S3).

All conversions were performed under the standard assumption of a linear association between PM concentration and the log-risk of the outcome. Following established meta-analytical methodology for dose-response data, reported estimates were converted to the common scale of per 10 µg/m^3^. For studies reporting a risk estimate per a different unit increment (e.g., per 1 µg/m^3^ or per 5 µg/m^3^), the log-risk estimate and its standard error were scaled accordingly. For studies reporting percentage changes per IQR, the estimate was first converted to a risk ratio before applying this scaling procedure [26, 27]. The standard errors for all converted estimates were derived to ensure correct weighting in the meta-analysis (Table S3). For the purpose of this synthesis, all measures of association (HR, OR, etc.) were treated as approximating the relative risk (RR), as the outcome of interest (diabetes-related mortality) is rare, making these measures numerically similar.

Meta-analyses were performed using both fixed-effect and random-effects models. The primary results are reported from the DerSimonian and Laird random-effects model to account for anticipated between-study heterogeneity. The fixed-effect model was applied for comparison. The pooled effect size is expressed as the relative risk (RR) of diabetes-associated mortality per 10 µg/m^3^ increase in PM exposure. Statistical heterogeneity was assessed using the Cochran’s Q test (p < 0.10 indicating significance) and the *I*^2^ statistic, which quantifies the percentage of variability due to heterogeneity rather than chance. *I*^2^ values of <25%, 25–75%, and >75% indicated low, moderate, and high heterogeneity, respectively. All analyses were conducted using Stata version 17.0 (StataCorp, College Station, TX, USA).

To explore potential sources of heterogeneity, we conducted subgroup and meta-regression analyses by: Type of PM, Study designs, region (Asia, Europe, Americas, others), Study population, Exposure type (long- or short-term), Study quality (high vs. moderate). Sensitivity analyses were performed by excluding one study at a time (leave-one-out approach) to assess the influence of individual studies on the overall pooled estimate. Potential publication bias was evaluated visually using funnel plots and statistically using Egger’s regression test and Begg’s rank correlation test.

## Results

### Search strategy and study selection

**Fig. 1:**
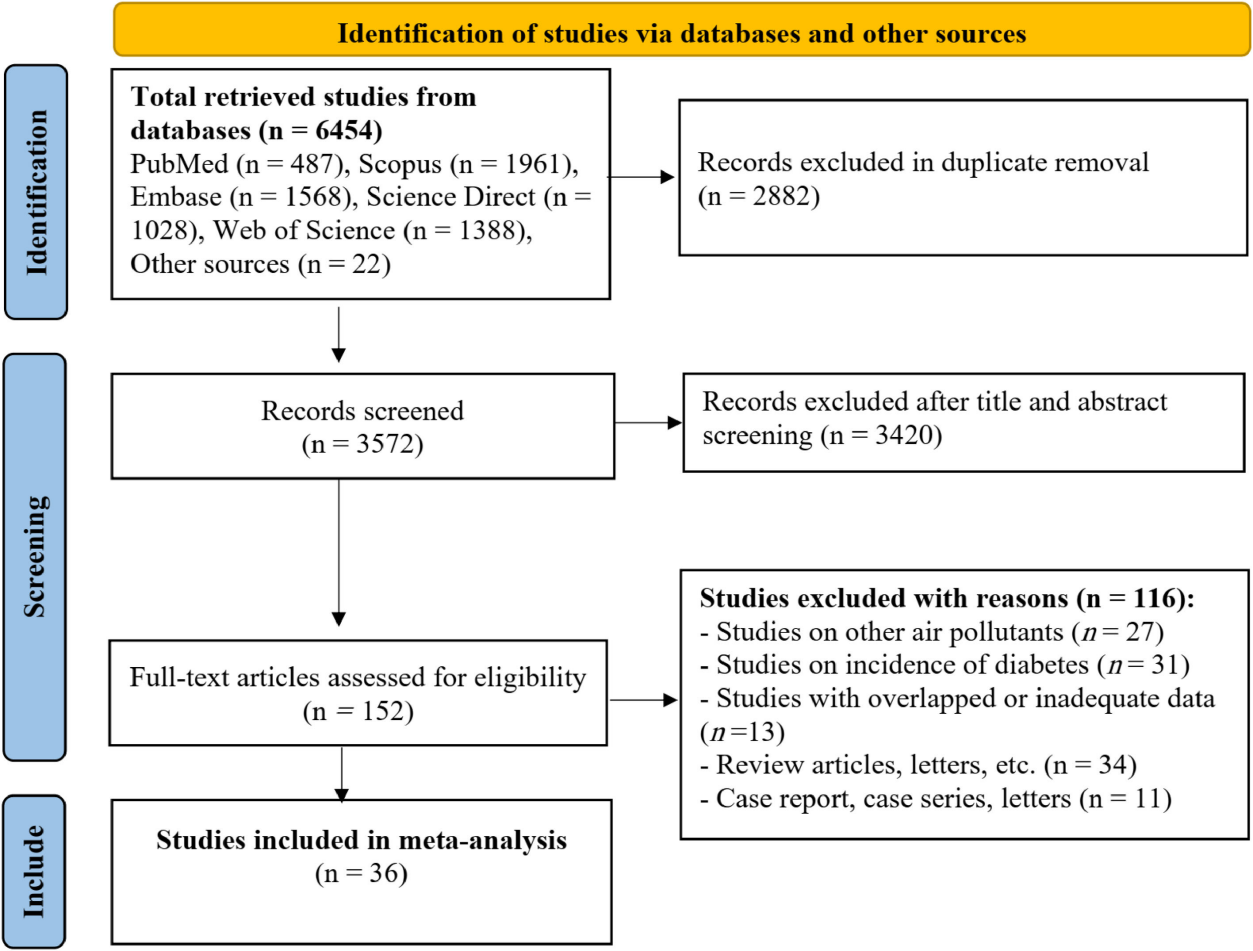
PRISMA flow diagram showing the literature search and study selection process.

The database search yielded 6454 records: 487 from PubMed, 1,961 from Scopus, 1,568 from Embase, 1,388 from Web of Science, 1028 from Science Direct, and 22 from other sources. After removing duplicates, and irrelevant studies, 152 full-text articles were assessed for eligibility, and 116 were excluded for the following reasons: focus on other air pollutants (n = 27), diabetes incidence rather than mortality (n = 31), overlapping or insufficient data (n = 13), review or commentary articles (n = 34), and case reports or case series (n = 11). Ultimately, 36 studies (59 datasets) published between 2001 and 2025 fulfilled all inclusion criteria and were included in the meta-analysis [28–63]. The detailed selection process is illustrated in Fig. 1. Main characteristics of included studies are presented in Table 1.

**Table 1:** Main features of eligible studies assessing the effects of PMs on risk of diabetes-related mortality

**Studies**	**short- or long- term exposure**	**Years Enrolled**	**Country**	**Study Design**	**Age Range (Years)**	**Type of PM**	**Converted to relative risk (RR) and 95% CI used in REM based on per 10 µg/m^3^ increment**	**Study quality**
Goldberg et al. (2001)	Short-term	1984–1993	Canada	TS	≥25	PM_2.5_	1.1 (1.02–1.18)	Moderate
Goldberg et al. (2001)	Short-term	1984–1993	Canada	TS	≥25	PM_10_	1.06 (1.01–1.11)	Moderate
Goldberg et al. (2001)	Short-term	1984–1993	Canada	TS	≥25	Predicted PM_2.5_	1.07 (1.02–1.14)	Moderate
Bateson et al. (2004)	Short-term	1988–1991	USA	CC	65+	PM_10_	1.01 (1.00–1.03)	High
Ostro et al. (2006)	Short-term	1999–2002	USA	TS	All ages	PM_2.5_	1.02 (1.01–1.04)	Moderate
Forastiere et al. (2008)	Short-term	1997–2004	Italy	CC	65+	PM_10_	1.01 (1.00–1.02)	High
Brook et al. (2013)	Long-Term	1991–2001	Canada	PC	≥25	PM_2.5_	1.49 (1.37–1.62)	High
Goldberg et al. (2013)	Short-term	1990–2003	Canada	TS	65+	PM_2.5_	1.02 (0.99–1.04)	Moderate
Zanobetti et al. (2014)	Short-term	1999–2010	USA	CC	>65	PM_2.5_	1.00 (1.00–1.01)	High
Pope et al. (2015)	Long-Term	1982–2004	USA	PC	≥30	PM_2.5_	1.13 (1.02–1.26)	High
Alessandrini et al. (2016)	Short-term	2006–2010	Italy	CC	≥65	PM_2.5_	1.02 (1.00–1.03)	High
Alessandrini et al. (2016)	Short-term	2006–2010	Italy	CC	≥65	PM_10_	1.02 (0.99–1.04)	High
Lim et al. (2018)	Long-Term	1995–2011	USA	PC	50–71	PM_2.5_	1.19 (1.03, 1.39)	High
Pinault et al. (2018)	Long-Term	2001–2011	Canada	PC	25–90	PM_2.5_	1.51 (1.39–1.65)	High
Pinault et al. (2018)	Long-Term	2001–2008	Canada	PC	25–90	PM_2.5_	1.52 (1.14–2.02)	High
Bowe et al. (2019)	Long-Term	2006–2016	USA	PC	Mean 64.1	PM_2.5_	1.36 (1.27–1.46)	High
Yang et al. (2020)	Short-term	2007–2013	China	TS	All ages	PM_10_	1.00 (1.00–1.01)	Moderate
Liu et al. (2020)	Long-Term	1990–2017	China	ES	25–94	PM_2.5_	1.60 (1.06–2.44)	Moderate
Liu et al. (2020)	Long-Term	1990–2017	USA	ES	25–94	PM_2.5_	1.22 (0.81–1.85)	Moderate
Liu et al. (2020)	Long-Term	1990–2017	China	ES	25–94	PM_2.5_	1.59 (0.91–2.77)	Moderate
Liu et al. (2020)	Long-Term	1990–2017	USA	ES	25–94	PM_2.5_	1.13 (0.70–1.82)	Moderate
Paul et al. (2020)	Long-Term	2001–2015	Canada	PC	35–85	PM_2.5_	1.10 (1.00–1.22)	High
Shan et al. (2020)	Long-Term	1998–2009	China	RC	Mean 44.12	PM_10_	2.26 (1.73–2.95)	High
So et al. (2020)	Long-Term	1993–2013	Denmark	PC	>44	PM_2.5_	2.19 (1.12–4.32)	High
Sui et al. (2020)	Short-term	2013–2018	China	TS	All ages	PM_2.5_	1.01 (0.99–1.02)	Moderate
Wu et al. (2021)	Short-term	2013–2019	China	TS	15–65, ≥65	PM_2.5_	1.01 (1.00–1.02)	Moderate
Wu et al. (2021)	Short-term	2013–2019	China	TS	15–65, ≥65	PM_10_	1.01 (1.00–1.02)	Moderate
Feng et al. (2021)	Long-Term	2010–2016	USA	PC	≥65	PM_2.5_	1.25 (1.13–1.38)	High
So et al. (2022)	Long-Term	2000–2017	Denmark	PC	≥30	PM_2.5_	1.21 (1.08–1.35)	High
Wu et al. (2022)	Long-Term	2006–2010	UK	PC	37–73	PM_2.5_	2.29 (1.89–2.78)	High
Wu et al. (2022)	Long-Term	2006–2010	UK	PC	37–73	PM_10_	1.47 (1.27–1.7)	High
Luo et al. (2023)	Long-Term	2006–2010	UK	PC	40–69	PM_2.5_	0.85 (0.55–1.44)	High
Yin et al. (2023)	Short-term	2013–2019	China	CC	All ages	PM_2.5_	1.01 (1.00–1.02)	Moderate
Yin et al. (2023)	Short-term	2013–2019	China	CC	All ages	PM_2.5–10_	1.01 (1.00–1.02)	Moderate
Gariazzo et al. (2023)	Short-term	2013–2015	Italy	TS	All ages	PM_2.5_	1.00 (0.91–1.11)	Moderate
Gariazzo et al. (2023)	Short-term	2013–2015	Italy	TS	All ages	PM_10_	1.02 (0.96–1.08)	Moderate
Aron et al. (2024)	Short-term	2016–2019	USA	CC	50–100	PM_2.5_	1.06 (1.00–1.12)	High
Guo et al. (2024)	Long-Term	2009–2015	China	PC	Mean 48.3	PM_2.5_	1.42 (1.21–1.67)	High
Guo et al. (2024)	Long-Term	2009–2015	China	PC	Mean 48.3	PM_1_	1.37 (1.02–1.87)	High
Guo et al. (2024)	Long-Term	2009–2015	China	PC	Mean 48.3	PM_1–2.5_	2.26 (1.70–3.01)	High
Moon et al. (2024)	Long-Term	2002–2019	South Korea	PC	50–79	PM_2.5_	1.34 (1.10–1.79)	High
Feng et al. (2024)	Short-term	2008–2011	China	TS	All ages	BC (PM_2.5_ component)	1.816 (1.21–2.72)	High
Feng et al. (2024)	Short-term	2008–2011	China	TS	All ages	NH_4_^+^ (PM_2.5_ component)	1.03 (0.89–1.19)	High
Feng et al. (2024)	Short-term	2008–2011	China	TS	All ages	NO_3_^−^ (PM_2.5_ component)	1.01 (0.93–1.10)	High
Feng et al. (2024)	Short-term	2008–2011	China	TS	All ages	OM (PM_2.5_ component)	1.06 (0.97–1.16)	High
Feng et al. (2024)	Short-term	2008–2011	China	TS	All ages	SO_4_^2−^ (PM_2.5_ component)	1.06 (0.92–1.24)	High
Wu et al. (2024)	Long-Term	2011–2013	China	PC	>20	PM_2.5_	1.95 (1.86, 2.04)	High
Wu et al. (2024)	Long-Term	2011–2013	China	PC	>20	PM_2.5–10_	0.96 (0.90, 1.02)	High
Zheng et al. (2024)	Long-Term	2006–2021	UK	PC	37–73	PM_2.5_	1.36 (1.26–1.46)	High
Zheng et al. (2024)	Long-Term	2006–2021	UK	PC	37–73	PM_10_	1.12 (1.05–1.20)	High
Hu et al. (2025)	Long-Term	2016–2023	China	PC	>40	PM_2.5_	1.06 (1.00–1.14)	High
Hu et al. (2025)	Long-Term	2016–2023	China	PC	>40	PM_2.5_	1.10 (1.05–1.16)	High
Hu et al. (2025)	Long-Term	2016–2023	China	PC	>40	PM_10_	1.02 (0.99–1.07)	High
Hu et al. (2025)	Long-Term	2016–2023	China	PC	>40	PM_10_	1.05 (1.02–1.08)	High
Oh et al. (2025)	Long-Term	2010–2019	South Korea	RC	≥65 years	PM_2.5_	1.05 (1.01–1.09)	High
Zhang et al. (2025)	Short-term	2016–2019	China	CC	NA	PM_2.5_	1.01 (1.00–1.02)	High
Wu et al. (2025)	Long-Term	2008–2017	USA	ES	Adults	PM_2.5_	1.15 (1.12–1.18)	Moderate
Yu et al. (2025)	Long-Term	2016–2022	China	PC	≥80	PM_2.5_	0.93 (0.84–1.02)	High
Yu et al. (2025)	Long-Term	2016–2022	China	PC	≥80	PM_10_	0.97 (0.92–1.03)	High

**Abbreviations:** TS, time series; PC, prospective cohort; RC, retrospective cohort; CC, case-crossover; ES, ecological study; NA, not available.

### Effects of PM_2.5_ on risk of diabetes-related mortality

#### Characteristics of included studies assessing effects of PM_2.5_ and risk of diabetes-related mortality

Forty-one datasets evaluated the association between PM_2.5_ exposure and diabetes-related mortality, comprising 25 long-term and 16 short-term cohorts across Asia (n = 18), North America (n = 16), and Europe (n = 7). The majority (n = 20) were cohort studies, complemented by time-series (n = 11) or case-crossover (n = 5) analyses and 5 datasets from ecological studies. Together, these studies encompassed over 70 million individuals and more than one million diabetes-attributed deaths. Most studies reported positive associations, with pooled estimates generally indicating a 5–15% increase in diabetes-related mortality per 10 µg/m^3^ rise in PM_2.5_. Confounding was typically controlled for age, sex, socioeconomic status, smoking, and comorbidities, while time-series studies further adjusted for seasonality and temperature. Overall, study quality was high (n = 29) to moderate (n = 12), and findings were consistent across diverse settings.

#### Results of meta-analysis on association between PM_2.5_ and risk of diabetes-related mortality

**Fig. 2:**
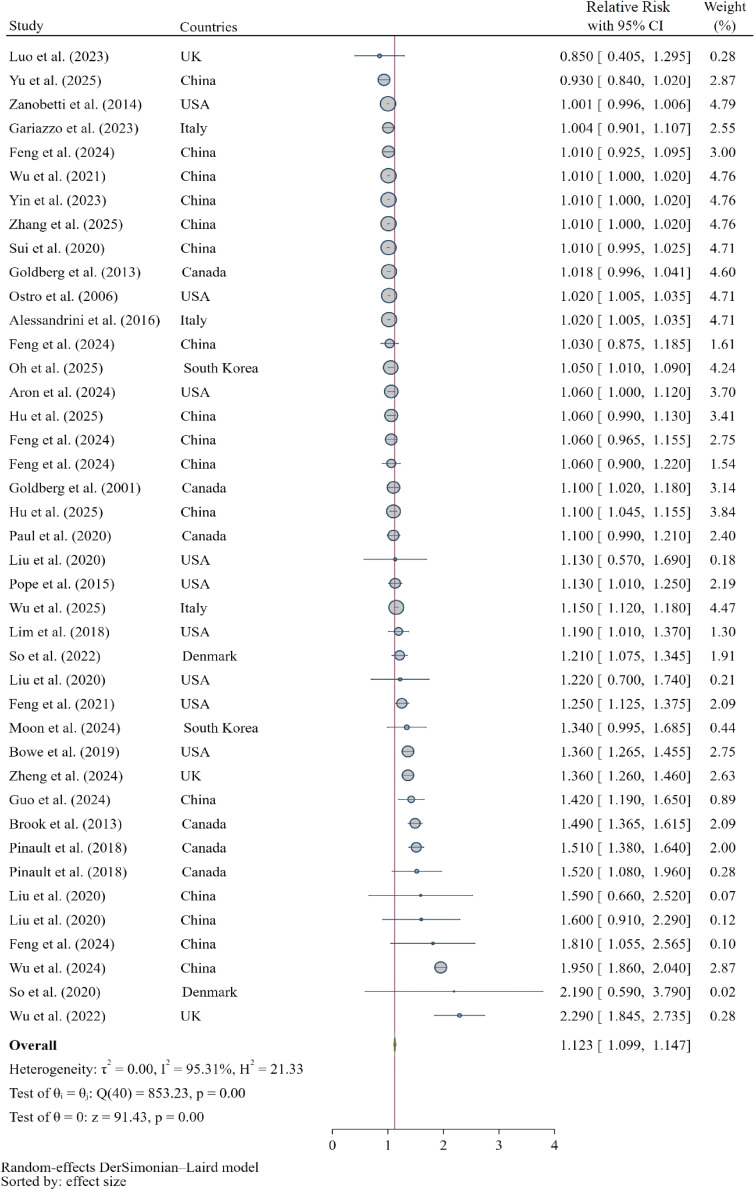
Forest plot for the association between PM_2.5_ exposure and risk of diabetes-related mortality

A total of 41 datasets from 28 eligible studies assessed the quantitative relationship between PM_2.5_ exposure and diabetes-related mortality. The pooled random-effects estimate demonstrated a significant positive association, with a 12.3% higher risk of diabetes-related death per 10 µg/m^3^ increase in PM_2.5_ (pooled RR = 1.123, 95% CI: 1.099–1.147; Fig. 2). Under a fixed-effects model, the corresponding estimate was smaller (RR = 1.014, 95% CI: 1.010–1.017), reflecting considerable between-study heterogeneity (*I*^2^ = 95.31%, Q = 853.2, τ^2^ = 0.00, Q-statistic p < 0.001; Table S4).

Subgroup analyses (Table 2) consistently indicated stronger associations in long-term exposure studies (RR = 1.296, 95% CI: 1.197–1.395) compared with short-term analyses (RR = 1.012, 95% CI: 1.006–1.019; p < 0.001 for subgroup difference). Prospective cohorts reported larger effects (RR = 1.327, 95% CI: 1.189–1.466) than time-series, case-crossover or ecological studies. Effect estimates were generally consistent across population types (general population RR = 1.140; specific patient cohorts RR = 1.114; p = 0.34), but varied modestly by geography—being highest in Europe (RR = 1.238, 95% CI: 1.056–1.420) and North America (RR = 1.157, 95% CI: 1.112–1.202), followed by East Asia (RR = 1.104, 95% CI: 1.064–1.143).

**Table 2:** Random effects subgroup meta-analyses for the associations between PM_2.5_ and diabetes-related mortality.

**Air pollutant**	**Number of ** **datasets**	**Risk estimate ** **(95% CI)**	**Test of group differences ** **(P-value)**	**Heterogeneity**	

				***I*^2^ (%)**	**Q**
**Published year**					
2001–2015	6	1.065 (1.027–1.104)	0.002	93.34	75.03
2015–2025	35	1.146 (1.113–1.179)		95.46	749.27
**Start year of data collection**					
1982–2000	13	1.087 (1.048–1.126)	0.013	87.41	95.28
2001–2010	18	1.204 (1.134–1.274)		92.96	241.50
≥2010	10	1.097 (1.053–1.141)		97.93	434.27
**Regions**					
***East Asia***	**18**	**1.104 (1.064–1.143)**	Regions P-value: 0.106 Countries P-value: <0.001	**96.30**	**460.05**
China	16	1.106 (1.064–1.148)		96.69	453.70
Korea	2	1.142 (0.878–1.407)		62.66	2.68
**Europe**	**7**	**1.238 (1.056–1.420)**		**92.86**	**84.06**
Italy	2	1.020 (1.005–1.035)		0.00	0.09
Denmark	2	1.362 (0.666–2.059)		30.12	1.43
UK	3	1.492 (0.867–2.118)		90.77	21.67
***North America***	**16**	**1.157 (1.112–1.202)**		**95.03**	**301.81**
USA	10	1.122 (1.071–1.173)		94.85	174.78
Canada	6	1.262 (1.086–1.437)		95.45	109.79
**Study Design**					
Prospective Cohort	19	1.327 (1.189–1.466)	<0.001	95.69	418.09
Retrospective cohort	1	1.050 (1.010–1.090)		-	-
Time-series	11	1.015 (1.006–1.024)		14.19	11.65
Case-crossover	5	1.010 (1.001–1.018)		64.97	11.42
Ecological	5	1.151 (1.122–1.181)		0.00	2.56
**Study population**					
General	31	1.140 (1.103–1.177)	0.339	91.79	365.46
Patients with specific disease	10	1.114 (1.075–1.152)		98.03	455.87
**Exposure type**					
Long-term exposure	25	1.296 (1.197–1.395)	<0.001	95.10	490.21
Short-term exposure	16	1.012 (1.006–1.019)		45.69	27.62
**Study quality**					
High quality	29	1.183 (1.144–1.223)	<0.001	96.32	761.15
Moderate quality	12	1.037 (1.014–1.060)		87.70	89.40

The robustness of this pooled estimate was confirmed through a leave-one-out sensitivity analysis. As detailed in Fig. S1, the sequential omission of any single study did not materially alter the overall conclusion, with the summary RR remaining statistically significant and ranging from 1.08 (95% CI: 1.06, 1.10) to 1.14 (95% CI: 1.11, 1.17). Visual inspection of the funnel plot (Fig. S2) suggested a relatively symmetric distribution of effect sizes around the summary estimate. However, the more sensitive Egger’s regression test provided statistical evidence of funnel plot asymmetry (Beta = 2.35, p < 0.001), indicating the potential presence of publication bias.

Univariable meta-regression identified exposure duration as a significant modifier of the effect size (β = 0.253, 95% CI: 0.054–0.452; p = 0.014). In a multivariable model adjusting for publication year, study start year, region, and study quality, exposure duration remained the only independent significant predictor of between-study heterogeneity. Other variables, including publication year, start year, study quality, and region, did not significantly explain between-study heterogeneity (adjusted R^2^ = 20.1%).

### Effects of PM_10_ on risk of diabetes-related mortality

#### Characteristics of included studies assessing effects of PM_10_ and risk of diabetes-related mortality

A total of 13 datasets from 12 eligible studies examined the relationship between PM_10_ exposure and diabetes-related mortality. These studies were geographically distributed across East Asia (mainly China, n = 6), Europe (Italy [n = 3], United Kingdom [n = 2]), and North America (United States [n = 1] and Canada [n = 1]), covering diverse environmental and demographic contexts. Six datasets assessed long-term exposure and seven datasets investigated effects of short-term exposure. The most datasets (n = 6) were cohort studies, followed by time-series (n = 4) and 3 datasets from case-crossover studies. Most studies derived exposure data from fixed monitoring networks or satellite-aided air-quality models, and adjusted for major covariates including age, sex, socioeconomic status, smoking, comorbidities, temperature, and seasonality. Overall methodological quality was high in nine studies and moderate in four, with principal limitations related to exposure misclassification and limited control of co-pollutants.

#### Results of meta-analysis on association between PM_10_ and risk of diabetes-related mortality

**Fig. 3:**
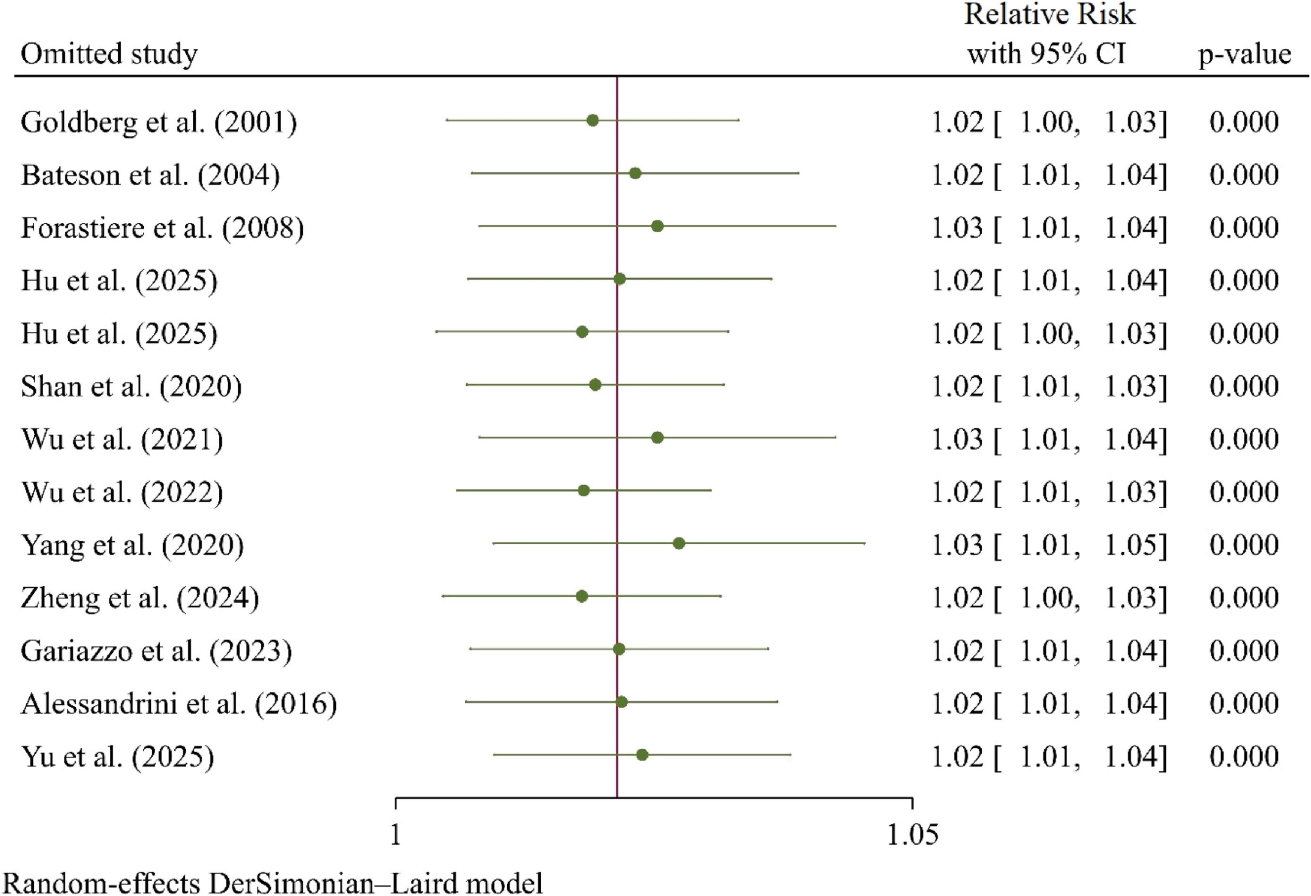
Forest plot for the association between PM_10_ exposure and risk of diabetes-related mortality

Pooled analysis of the 13 datasets showed a statistically significant positive association between PM_10_ exposure and diabetes-related mortality. The random-effects summary estimate indicated a 2.1% increase in mortality risk per 10 µg/m^3^ increment in PM_10_ (pooled RR = 1.021, 95% CI 1.007–1.035; I^2^ = 81.7%, Q = 65.5; Fig. 3). The corresponding fixed-effects model produced a similar direction of effect (RR = 1.007, 95% CI 1.003–1.010; Table S4).

Subgroup analyses (Table 3) demonstrated that the association was stronger in long-term exposure studies (RR = 1.095, 95% CI 1.016–1.175) compared with short-term studies (RR = 1.010, 95% CI 1.002–1.017; p = 0.035). Prospective cohorts yielded higher pooled estimates (RR = 1.067, 95% CI 1.002–1.133) than time-series (RR = 1.009, 95% CI 0.996–1.021) or case-crossover (RR = 1.012, 95% CI 1.004–1.020) analyses. Effect estimates were relatively consistent across geographic regions, though slightly larger in Europe (RR = 1.050, 95% CI 1.006–1.094) than in East Asia (RR = 1.014, 95% CI 0.994–1.034) or North America (RR = 1.031, 95% CI 0.989–1.073).

**Table 3:** Random effects subgroup meta-analyses for the associations between PM_10_ and diabetes-associated mortality

**Air pollutant**	**Number of datasets**	**Risk estimate ** **(95% CI)**	**Test of group differences ** **(P-value)**	**Heterogeneity**	

				***I*^2^ (%)**	**Q**
**Published year**					
2001–2015	3	1.016 (1.001–1.030)	0.442	47.32	3.80
2015–2025	10	1.025 (1.005–1.046)		84.75	59.00
**Start year of data collection**					
1982–2000	4	1.026 (0.993–1.059)	0.370	84.89	19.85
2001–2010	4	1.059 (1.005–1.113)		89.94	29.82
≥2010	5	1.017 (0.995–1.039)		53.97	8.69
**Regions**					
***East Asia***	**6**	**1.014 (0.994–1.034)**	Regions P-value: 0.301 Countries P-value: 0.210	**83.42**	**30.16**
China	6	1.014 (0.994–1.034)		83.42	30.16
Korea	-	-		-	-
**Europe**	**5**	**1.050 (1.006–1.094)**		84.51	25.82
Italy	3	1.012 (1.002–1.021)		0.00	0.59
UK	2	1.280 (0.938–1.622)		88.98	9.08
***North America***	**2**	**1.031 (0.989–1.073)**		65.01	2.86
USA	1	1.015 (1.000–1.030)		-	-
Canada	1	1.060 (1.010–1.110)		-	-
**Study Design**					
Prospective Cohort	5	1.067 (1.002–1.133)	<0.001	85.37	27.34
Retrospective cohort	1	2.260 (1.650–2.870)		-	-
Time-series	4	1.009 (0.996–1.021)		61.18	7.73
Case-crossover	3	1.012 (1.004–1.020)		0.00	0.68
**Study population**					
General	10	1.031 (1.008–1.053)	0.131	85.55	62.27
Patients with specific disease	3	1.012 (1.004–1.020)		0.00	0.68
**Exposure type**					
Long-term exposure	6	1.095 (1.016–1.175)	0.035	88.29	42.70
Short-term exposure	7	1.010 (1.002–1.017)		50.66	12.16
**Study quality**					
High quality	9	1.033 (1.006–1.059)	0.104	83.89	49.65
Moderate quality	4	1.009 (0.996–1.021)		61.18	7.73

The leave-one-out sensitivity analysis (Fig. S3) confirmed the robustness of the overall positive finding. The sequential removal of any single study did not alter the statistical significance of the summary estimate, which remained stable, ranging from RR = 1.02 (95% CI: 1.00, 1.03) to RR = 1.03 (95% CI: 1.01, 1.05). Notably, the exclusion of the two studies with the largest effect sizes (Shan et al., 2020 and Wu et al., 2022) did not nullify the association, indicating that the positive finding is not solely driven by extreme outliers. However, Egger’s regression test provided strong statistical evidence of publication bias (Beta = 2.52, Standard Error = 0.462, p < 0.001; Fig. S4).

### Effects of other PMs and risk of diabetes-related mortality

Beyond PM_2.5_ and PM_10_, several studies investigated the association of other particulate matter (PM) size fractions with diabetes-related mortality. A single study utilizing a predicted PM_2.5_ exposure model reported a significant 7% increase in mortality risk per 10 µg/m^3^ increment (RR = 1.07, 95% CI: 1.01–1.13). In contrast, evidence for coarser particles (PM_2.5–10_) from two datasets indicated a null association (RR = 0.99, 95% CI: 0.95–1.04; *I*^2^ = 61.5%), suggesting a minimal role for coarse fractions in diabetes mortality. Conversely, finer and ultrafine fractions demonstrated more pronounced effects. While based on a single study, PM_1_ was associated with a non-significant but elevated risk (RR = 1.37, 95% CI: 0.95–1.80). Most notably, exposure to PM_1–2.5_ was associated with a markedly higher risk estimate in a single study (RR = 2.26, 95% CI: 1.61–2.92). This finding, while intriguing, is preliminary and requires confirmation from independent studies.

## Discussion

In this systematic review and meta-analysis of 36 studies, we found that long- and short-term exposure to ambient PMs was positively associated with diabetes-related mortality. The pooled random-effects estimate for PM_2.5_ indicated a 12.3% increase in diabetes-attributed mortality per 10 µg/m^3^ increase. PM_10_ exposure was also associated with 2.1% increased risk. These results were robust in sensitivity analyses but observed with high between-study heterogeneity. Evidence from limited studies on smaller particle fractions (PM_1_ and PM_1–25_) suggests potentially stronger effects of ultrafine and fine components, supporting the notion that smaller particles exert greater biological impact. Subgroup analyses indicated that long-term exposure and prospective study designs yielded more pronounced associations, suggesting a cumulative effect of chronic PMs exposure on diabetes mortality risk. Regional estimates varied modestly (largest in Europe and North America), but exposure duration was the only independent predictor of between-study heterogeneity in multivariable meta-regression (β ≈ 0.253; p = 0.014).

While several meta-analyses have assessed the effect of air pollutions and PMs on diabetes incidence [7, 8, 64], quantitative syntheses focusing specifically on diabetes-related mortality remain scarce. Our findings can be directly compared to the seminal 2014 meta-analysis by Li et al. [13], which, to our knowledge, is the only previous work to quantitatively pool risk estimates for this specific outcome. The pooled RR for PM_2.5_-associated diabetes mortality reported by Li et al. was 1.123 (95% CI: 1.036–1.217) per 10 µg/m^3^, an estimate identical to ours (RR = 1.123, 95% CI: 1.099–1.147). This remarkable consistency, despite a near-decade difference in the literature covered, suggests a stable and robust association across an expanding evidence base. However, our analysis for PM_10_ yielded a notably stronger association (RR = 1.021, 95% CI: 1.007–1.035) compared to the earlier estimate (RR = 1.008, 95% CI: 1.004–1.013) [13]. This divergence likely reflects the evolution of the evidence; our synthesis incorporated more than three times the number of datasets for PM_10_, including several large-scale cohort studies published after 2014 that reported higher risk estimates [49, 59, 63]. Furthermore, differences in the included studies’ geographic distribution, exposure assessment methodologies (e.g., increased use of satellite-based models), and the proportion of long-term exposure studies may also account for the variation in the pooled effect size.

The association observed for PM_10_ in the present analysis was weaker than that for PM_2.5_, aligning with the hypothesis that finer particles exert greater toxicological potency due to their larger surface area, deeper pulmonary deposition, and enhanced capacity to translocate into the systemic circulation [65, 66]. This size-dependent gradient has been consistently reported in prior research on cardiovascular and metabolic outcomes and supports the biological plausibility of the observed findings [67, 68].

A pivotal finding from our subgroup and meta-regression analyses is the significantly stronger association observed for long-term PM exposure compared to short-term effects. For PM_2.5_, the risk estimate for long-term exposure (RR = 1.296) was substantially larger than for short-term exposure (RR = 1.012). This pattern underscores the cumulative impact of chronic PMs exposure on metabolic health. Biologically, prolonged inhalation of fine particles induces sustained systemic inflammation and oxidative stress [11, 14]. These processes can impair insulin signaling, promote endothelial dysfunction, and exacerbate atherosclerosis, thereby accelerating the progression of diabetes complications—such as cardiovascular and renal disease—and ultimately elevating mortality risk [14]. The stronger effects seen in prospective cohorts further support the notion that the deleterious metabolic consequences of PM exposure accumulate over time, leading to a greater mortality burden.

A primary consideration in interpreting our findings is the substantial between-study heterogeneity, as indicated by the high *I*^2^ statistics (95.3% for PM_2.5_ and 81.7% for PM_10_). This heterogeneity, while common in environmental meta-analyses, underscores the influence of methodological and population differences across the included studies and necessitates a cautious interpretation of the pooled point estimates [8]. Our subgroup and meta-regression analyses were instrumental in exploring potential sources of this heterogeneity. The most significant modifier was the duration of exposure, with long-term studies showing consistently and substantially stronger associations than short-term studies. This is methodologically coherent, as cohort designs capturing chronic exposure account for the cumulative pathological processes leading to death [11], whereas time-series and case-crossover studies capture acute exacerbations in already vulnerable individuals. Furthermore, variability in exposure assessment methods—ranging from central monitoring stations to sophisticated satellite-based models and land-use regression—could contribute to exposure misclassification to varying degrees, influencing the observed effect sizes [42, 49]. Geographic and climatic differences, along with variations in the chemical composition of PMs across regions, also likely play a role, as suggested by the modest variation in risk estimates between North America, Europe, and Asia [39, 69]. Finally, differences in the underlying population characteristics, such as age structure, genetic susceptibility, and prevalence of comorbidities, as well as the extent of adjustment for key confounders like socioeconomic status, smoking, and BMI, further contribute to the heterogeneous landscape of the evidence base [38, 58].

Furthermore, contextual factors beyond exposure assessment may contribute to regional differences in risk estimates. Variations in healthcare access, quality of diabetes management, and cause-of-death certification practices across different countries and regions could influence the recorded outcome of diabetes-related mortality. For instance, in settings with less robust primary care, the contribution of diabetes to mortality may be under-ascertained, potentially attenuating observed associations. Conversely, regions with advanced diagnostic capabilities and detailed death registry data might more accurately identify diabetes as a contributing cause. Differences in competing mortality risks (e.g., prevalence of infectious diseases, trauma) could also affect the baseline risk and observed strength of association. While our meta-regression did not identify region as an independent, statistically significant predictor after accounting for exposure duration, these systemic factors remain important considerations when translating these pooled estimates to specific public health contexts.

The interpretation of our results must also consider the potential for publication bias. While funnel plots appeared roughly symmetrical, the more sensitive Egger’s regression test provided statistical evidence of asymmetry for both PM_2.5_ (p < 0.001) and PM_10_ (p < 0.001). This suggests a potential bias where small studies with null or non-significant results may be missing from the published literature. The implication is that the overall pooled risk estimates reported in our meta-analysis might be somewhat inflated. Therefore, the true population effect size for the association between PMs and diabetes-related mortality could be smaller, though highly likely to remain positive and statistically significant given the magnitude and consistency of the findings across a large number of studies, including major cohorts. This potential bias does not negate the positive association but suggests a need for caution in over-interpreting the precise magnitude of the point estimates.

The observed association between PMs exposure and diabetes-related mortality is considered biologically plausible and supported by a growing body of experimental and epidemiological evidence [70, 71]. As outlined in the introduction, the primary pathways through which particulate matter, particularly PM_2.5_, is thought to contribute to diabetes pathogenesis and mortality involve systemic inflammation, oxidative stress, and endothelial dysfunction [72]. Systemic inflammation is one of the principal mechanisms linking particulate exposure to diabetes progression. Inhaled PM_2.5_ activates pulmonary macrophages and epithelial cells, releasing pro-inflammatory cytokines such as interleukin-6 (IL-6), tumor necrosis factor-α (TNF-α), and C-reactive protein (CRP), which in turn contribute to insulin resistance and endothelial dysfunction [14, 65]. Chronic exposure maintains a low-grade inflammatory state that accelerates vascular injury and impairs glucose metabolism. Oxidative stress represents another critical pathway. Transition metals, polycyclic aromatic hydrocarbons, and other redox-active components of PM can generate reactive oxygen species (ROS), leading to mitochondrial dysfunction, β-cell impairment, and lipid peroxidation [73, 74]. These oxidative processes not only disturb insulin signaling but also exacerbate cardiovascular comorbidities, thereby increasing mortality risk among individuals with diabetes [73, 74]. Additionally, PMs exposure has been implicated in autonomic imbalance and epigenetic alterations [75]. Chronic exposure can reduce heart rate variability and disrupt neuroendocrine control of glucose homeostasis, while epigenetic changes in genes regulating inflammation and insulin signaling (e.g., methylation of IL6 or TNF promoters) have been observed in long-term exposure contexts [76]. The stronger associations observed for long-term exposure in this meta-analysis align with these mechanistic pathways, as cumulative oxidative and inflammatory stress can amplify metabolic dysregulation over time. The steeper exposure-response gradient observed for PM_2.5_ compared to PM_10_ aligns with the well-established size-dependent toxicity of PMs. The smaller aerodynamic diameter of PM_2.5_ allows for greater alveolar deposition and translocation into the bloodstream, presenting a larger surface area per unit mass for the adsorption and delivery of toxic compounds directly to systemic targets. In contrast, PM_10_ particles are largely deposited in the upper airways and are less likely to provoke the same degree of systemic metabolic disturbance [77, 78].

Beyond these direct biological mechanisms, indirect pathways may also contribute to the observed association. High levels of ambient PM often lead to advisory warnings and behavioral changes, such as reduced time spent outdoors. This decrease in outdoor physical activity, coupled with potential disruptions to daily routines, could negatively influence glycemic control and cardiovascular health in individuals with diabetes over time. While the relative contributions of direct toxicological effects versus indirect behavioral mediators are challenging to disentangle in population studies, the stronger associations observed for long-term exposure—which reflects a chronic, background level of pollution less likely to drive sustained behavioral avoidance—suggest that direct biological effects play a primary role. Nonetheless, the potential for indirect pathways, especially in the context of acute, high-concentration episodes, should be considered in a holistic understanding of PM’s impact on diabetes outcomes.

Intriguingly, our analysis of limited data on smaller fractions suggests that particles in the PM_1–2.5_ range may be associated with a markedly elevated risk. However, this result is based on a single study and must be interpreted with considerable caution. The PM_1–2.5_ fraction is often rich in combustion-derived components, such as transition metals and organic carbon, which are highly reactive and potent inducers of oxidative stress [79]. Furthermore, this size fraction may represent a “sweet spot” for pulmonary deposition and systemic bioavailability, being small enough to reach the deep lung yet large enough to carry a significant toxic payload. While biologically plausible, as this fraction may carry a high burden of combustion-derived toxicants, the magnitude of the observed association (RR > 2) raises the possibility of methodological factors specific to that study, such as the particular pollution mix, exposure model, or population characteristics, contributing to an overestimation. This underscores that the health impacts of PMs are not uniform and that the specific composition and sources of fine particles are critical determinants of their metabolic toxicity [80, 81]. Robust conclusions regarding the PM_1–2.5_ fraction await further investigation.

This study’s key strengths include its rigorous adherence to PRISMA/MOOSE guidelines, a comprehensive and updated literature search, and the inclusion of a large body of evidence from over 70 million individuals, providing substantial statistical power. The stability of our findings was confirmed through extensive sensitivity and subgroup analyses. However, our results must be interpreted in the context of certain limitations. As previously discussed, the high degree of between-study heterogeneity and the statistical evidence of publication bias are important caveats, suggesting that the pooled effect sizes should be considered as the best available, albeit potentially inflated, estimates. A fundamental limitation inherent in all observational meta-analyses is the potential for residual confounding in the original studies; despite most studies adjusting for key variables like age, sex, and smoking, unmeasured or imperfectly measured factors could still influence the associations. Additionally, the reliance in many included studies on area-level exposure estimates derived from monitoring networks or models, rather than personal exposure measurements, can lead to exposure misclassification. This approach assumes a correlation between ambient concentration and personal inhalation, which may be attenuated by factors such as time spent indoors, use of air filtration, or individual mobility patterns. While this misclassification is typically non-differential and would likely bias results toward the null, it may still contribute to the observed heterogeneity and imprecision. Furthermore, differences in exposure assessment methodologies (e.g., central monitor vs. satellite-based model vs. land-use regression) across studies represent an important source of between-study variability, as noted in our discussion of heterogeneity. Furthermore, the inclusion of ecological studies, while broadening the evidence base, introduces the potential for ecological fallacy, where associations observed at the group level may not accurately reflect individual-level risks. We mitigated this by conducting subgroup analyses by study design and interpreting findings from ecological studies with appropriate caution.

## Conclusion

In summary, this comprehensive and updated meta-analysis provides robust evidence that exposure to ambient PMs, especially fine PM_2.5_, is a significant risk factor for diabetes-related mortality. The stronger association observed with long-term exposure underscores the cumulative metabolic damage inflicted by chronic air pollution. These findings highlight a substantial and modifiable environmental contributor to the global burden of diabetes mortality. Consequently, our results lend strong support to the critical importance of establishing and enforcing more stringent air quality standards. Such public health and policy interventions are imperative to mitigate the metabolic health burden of air pollution and reduce premature deaths from diabetes worldwide.

## Supplementary information

**Additional file 1:** **Table S1.** Search strategy in databases. **Table S2.** Adjusted confounders in studies examining particulate matter exposure and risk of diabetes-associated mortality. **Table S3.** Details of Effect Estimate Conversion for Meta-Analysis. **Table S4.** Random and fixed effects meta-analyses for the associations between air pollutions and risk of diabetes-related mortality. **Table S5.** meta regression between study variables and risk of diabetes-associated mortality due to PM_2.5_. **Figure S1.** Sensitivity analysis for the association between PM_2.5_ exposure and risk of diabetes-related mortality. **Figure S2:** Funnel plot for publication bias in PM_2.5_ and diabetes mortality studies. **Figure S3.** Sensitivity analysis for the association between PM_10_ exposure and risk of diabetes-related mortality. **Figure S4:** Funnel plot for publication bias in PM_10_ and diabetes mortality studies.

## Declarations

### Data availability

Data used in this study are available in main document or supplementary files. Further data will be made available on request.
