# Supplementary material for: Exposure to particulate matters and risk of diabetes-related mortality: a systematic review and meta-analysis
Source: Environ Health Prev Med. 2026 Mar 7;31:18. doi: 10.1265/ehpm.25-00424 (PMC12981974; doi:10.1265/ehpm.25-00424)
Supplement: Supplementary file 1 — Additional file 1: Table S1. Search strategy in databases. Table S2. Adjusted confounders in studies examining particulate matter exposure and risk of diabetes-associated mortality. Table S3. Details of Effect Estimate Conversion for Meta-Analysis. Table S4. Random and fixed effects meta-analyses for the associations between air pollutions and risk of diabetes-related mortality. Table S5. meta regression between study variables and risk of diabetes-associated mortality due to PM2.5. Figure S1. Sensitivity analysis for the association between PM2.5 exposure and risk of diabetes-related mortality. Figure S2: Funnel plot for publication bias in PM2.5 and diabetes mortality studies. Figure S3. Sensitivity analysis for the association between PM10 exposure and risk of diabetes-related mortality. Figure S4: Funnel plot for publication bias in PM10 and diabetes mortality studies. [file ehpm-31-018-s001.docx]

**Table S1**. Search strategy in databases

| **Database** | **Keywords** | **Results** |
| --- | --- | --- |
| PubMed | ("Particulate Matter"[Mesh] OR "Air Pollution"[Mesh] OR particulate matter[tiab] OR "particulate matter"[tiab] OR PM2.5[tiab] OR PM2_5[tiab] OR PM10[tiab] OR "PM 2.5"[tiab] OR "PM 10"[tiab] OR "fine particulate"[tiab] OR "coarse particulate"[tiab] OR "black carbon"[tiab] OR soot[tiab] OR "diesel exhaust"[tiab] OR "traffic-related air pollution"[tiab] OR TRAP[tiab] OR aerosols[tiab] OR pollut*[tiab]) AND ("Diabetes Mellitus"[Mesh] OR "Diabetes Complications"[Mesh] OR diabetes[tiab] OR "diabetes mellitus"[tiab] OR "diabetes-related"[tiab] OR "diabetes associated"[tiab] OR "diabetic"[tiab]) AND (mortality[tiab] OR mortalit*[tiab] OR death[tiab] OR "cause-specific mortality"[tiab] OR "diabetes mortality"[tiab] OR "diabetes-related death"[tiab] OR "diabetic death"[tiab]) | 487 |
| Scopus | (TITLE-ABS-KEY("particulate matter" OR "particulate" OR PM2.5 OR PM2_5 OR PM10 OR "fine particulate" OR "coarse particulate" OR "black carbon" OR soot OR "diesel exhaust" OR "traffic-related air pollution" OR TRAP OR aerosol* OR pollut*) AND TITLE-ABS-KEY(diabetes OR "diabetes mellitus" OR diabetic OR "diabetes-related" OR "diabetes associated") AND TITLE-ABS-KEY(mortality OR mortalit* OR death OR "diabetes mortality" OR "diabetes-related death" OR "cause-specific mortality")) | 1961 |
| Embase | ('particulate matter'/exp OR 'air pollution'/exp OR "particulate matter":ti,ab OR PM2.5:ti,ab OR PM2_5:ti,ab OR "PM 2.5":ti,ab OR PM10:ti,ab OR "PM 10":ti,ab OR "fine particulate":ti,ab OR "coarse particulate":ti,ab OR "black carbon":ti,ab OR soot:ti,ab OR "diesel exhaust":ti,ab OR "traffic-related air pollution":ti,ab OR TRAP:ti,ab OR aerosol*:ti,ab OR pollut*:ti,ab) AND ('diabetes mellitus'/exp OR 'diabetes complication'/exp OR diabetes:ti,ab OR "diabetes mellitus":ti,ab OR diabetic:ti,ab OR "diabetes-related":ti,ab OR "diabetes associated":ti,ab) AND (mortality:ti,ab OR mortalit*:ti,ab OR death:ti,ab OR "cause-specific mortality":ti,ab OR "diabetes mortality":ti,ab OR "diabetes-related death":ti,ab OR "diabetic death":ti,ab) | 1568 |
| Science direct | ("particulate matter" OR PM2.5 OR PM10 OR "fine particulate") AND (diabetes OR "diabetes mellitus" OR diabetic) AND (mortality OR mortalit* OR death) | 1028 |
| WOS | ("particulate matter" OR particulate OR PM2.5 OR PM2_5 OR "PM 2.5" OR PM10 OR "PM 10" OR "fine particulate" OR "coarse particulate" OR "black carbon" OR soot OR "diesel exhaust" OR "traffic-related air pollution" OR TRAP OR aerosol* OR pollut*) AND (diabetes OR "diabetes mellitus" OR diabetic OR "diabetes-related" OR "diabetes associated") AND (mortality OR mortalit* OR death OR "diabetes mortality" OR "diabetes-related death" OR "cause-specific mortality") | 1388 |

**Table S2.** Adjusted confounders in studies examining particulate matter exposure and risk of diabetes-associated mortality

| **Study** | **Particulate matter** | **Adjusted confounding factors** |
| --- | --- | --- |
| Brook et al. (2013) | PM₂.₅ | Age, sex, aboriginal ancestry, visible minority, marital status, education, employment status, occupation, low-income cutoff, community size, contextual covariates (% adults without high school diploma, % unemployed, % in lowest income quintile at census tract and division levels) |
| Goldberg et al. (2001) | PM₂.₅  PM₁₀  Predicted PM₂.₅ | Seasonal trends, weather variables, calendar year |
| Goldberg et al. (2013) | PM₂.₅ | Long-term temporal trends, daily maximum temperature, day of week |
| Ostro et al. (2006) | PM₂.₅ | Time trends, seasonality, temperature, humidity, day of week |
| Zanobetti et al. (2014) | PM₂.₅ | Day of week, temperature |
| Bateson et al. (2004) | PM₁₀ | Temperature, humidity, barometric pressure, day of week |
| Forastiere et al. (2008) | PM₁₀ | Influenza epidemics, population changes, holidays, barometric pressure, apparent temperature, time trends, day of week |
| Aron et al. (2024) | PM₂.₅ | Daily minimum temperature, daily minimum relative humidity, daily precipitation, holiday status |
| Bowe et al. (2019) | PM₂.₅ | Age, race, sex, smoking status, population density, Area Deprivation Index, rural residence, access to healthy food, exercise opportunities, excessive drinking |
| Guo et al. (2024) | PM₂.₅ | Age, sex, ethnicity, education, marital status, medical insurance, exercise frequency, smoking status, NDVI |
| Hu et al. (2025) | PM₂.₅  PM₁₀ | Age, sex, marriage, occupation, presence of respiratory diseases or cardiovascular diseases, urban or rural residence, number of medical and technical personnel per 1000 population, real GDP per capita, percentage of high school graduates, long-term temperature variability, long-term exposure to relative humidity |
| Lim et al. (2018) | PM₂.₅ | Age, sex, region, race, education, marital status, BMI, alcohol, smoking, diet (fat, vegetable, fruit consumption), census tract median income, census tract % with less than high school education |
| Liu et al. (2020) | PM₂.₅ | age-period-cohort model, sex stratification |
| Luo et al. (2023) | PM₂.₅ | Age, sex, BMI, race, education, Townsend Deprivation Index, recruitment center, alcohol drinking, smoking, diet, pre-existing hypertension |
| Moon et al. (2024) | PM₂.₅ | Age, sex, income, disability, comorbidities (CHF, cerebrovascular disease, COPD, DM, hypertension, cancer), district-level population density, basic security pension, unmet medical needs, region |
| Oh et al. (2025) | PM₂.₅ | Gender, age, type of insurance enrollment, income level, underlying disease, total population (district-level), proportion of older adults (≥65 years, district-level), education level (district-level), annual mean temperature (district-level), rainfall (district-level), smoking rate (district-level), and strata (region) |
| Paul et al. (2020) | PM₂.₅ | Age, sex, area-level SES, region classification, healthcare access, ethnicity, comorbidities |
| Pinault et al. (2018) | PM₂.₅ | Age, sex, population center size, airshed, aboriginal identity, visible minority status, educational attainment, labor force status, income adequacy quintile, neighborhood covariates (% unemployed, % without high school diploma, % low-income families) |
| Shan et al. (2020) | PM_10_ | Age, gender, BMI, smoking status, education level, personal income, exercise, marital status, occupational PM exposure, dietary habits (meat, poultry, seafood, fruit, vegetable, alcohol consumption) |
| So et al. (2020) | PM₂.₅ | Age, BMI, smoking status, alcohol consumption, physical activity, fatty meat consumption, working status, marital status, use of hormone therapy, road traffic noise |
| Feng et al. (2024) | PM_2.5_ components (e.g., BC, NH₄⁺, NO₃⁻, OM, SO₄²⁻) | Temperature, humidity, pressure, PM10, NO2, SO2 |
| Wu et al. (2021) | PM₂.₅, PM₁₀ | Day of week, time trends, temperature, relative humidity |
| Wu et al. (2022) | PM₂.₅  PM₁₀ | Age, sex, ethnicity, residential area, smoking status, healthy diet, physical activity, family history of diabetes |
| Wu et al. (2024) | PM₂.₅, PM_2.5-10_ | Age, sex, BMI, family history of diabetes, drinking status, smoking status, physical activity, employment, medication compliance, comorbidities, GDP, doctors per 10,000 |
| Yang et al. (2019) |  | Temperature, humidity, atmospheric pressure, day of week, holidays, seasonality, long-term trend |
| Yin et al. (2023) | PM₂.₅, PM_2.5-10_ | Temperature, relative humidity, holidays, day of week, seasonality |
| Zhang et al. (2025) | PM₂.₅ | Temperature, relative humidity, holiday |
| Zheng et al. (2024) | PM₂.₅, PM₁₀ | Age, sex, race, income, residence, family history of diabetes, smoking, alcohol, physical activity, BMI, multivitamin, DDS, CVD, cancer, hypertension, hyperlipidemia |
| Zúñiga et al. (2016) | PM₁₀ | Seasonal trends, weather variables, influenza cases |
| Gariazzo et al. (2023) | PM₂.₅, PM₁₀ | Long-term seasonal time trends, day of week, province-specific air temperature (lag 0-1 and 1-6 modeled), holidays, summer population decrease, influenza epidemics, municipality×year×month×day interactions (time trend adjustment) |
| Feng et al. (2021) | PM₂.₅ | Age, sex, race, ZIP code-level SES, cause of kidney failure, smoking status, BMI, nephrology care status |
| Alessandrini et al. (2016) | PM₂.₅, PM₁₀ | Influenza epidemics, population changes, holidays, barometric pressure, apparent temperature, time trends, day of week |
| Pope et al. (2014) | PM₂.₅ | Age, sex, race, smoking, second-hand smoke, occupational exposure, marital status, education, BMI, alcohol, diet |
| Sui et al. (2020) | PM₂.₅ | Temperature, relative humidity, day of week, seasonal trends |
| Wu et al. (2025) | PM₂.₅ | Temperature, GDP per capita, county and year fixed effect |
| Yu et al. (2025) | PM₂.₅, PM₁₀ | Age, sex, marital status, occupation, type of residence, smoking, drinking |

**Table S3.** Details of Effect Estimate Conversion for Meta-Analysis.

| **Study and First Author** | **Pollutant** | **Reported Effect Estimate (e.g., HR, RR, IQR) and 95% CI** | **Increment as Originally Reported (e.g., "per 5 µg/m)** | **Converted to relative risk (RR) and 95% CI used in REM^🞸^ based on per 10 µg/m³ increment** |
| --- | --- | --- | --- | --- |
| Brook et al. (2013) | PM₂.₅ | HR, 1.49 (1.37–1.62) | per 10 µg/m³ | 1.49 (1.37–1.62) |
| Goldberg et al. (2001) | PM₂.₅ | MPC, 12.03 (3.01–21.84) | per 12.51 µg/m³ (IQR) | 1.10 (1.02–1.18) |
| Goldberg et al. (2001) | PM₁₀ | MPC, 13.20 (2.69–24.79) | per 21.32 µg/m³ (IQR) | 1.06 (1.01–1.11) |
| Goldberg et al. (2001) | Predicted PM₂.₅ | MPC, 7.59 (2.36–13.09) | per 9.50 µg/m³ (IQR) | 1.07 (1.02–1.14) |
| Goldberg et al. (2013) | PM₂.₅ | MPC, 1.83 (-0.53–4.25) | per 6.88 µg/m³ (IQR) | 1.02 (0.99–1.04) |
| Ostro et al. (2006) | PM₂.₅ | MPC, 2.2 (0.6–3.9) | per 10 µg/m³ | 1.02 (1.01–1.04) |
| Zanobetti et al. (2014) | PM₂.₅ | PI, 0.76 (0.39–1.12) | per 10 µg/m³ | 1.00 (1.00–1.01) |
| Bateson et al. (2004) | PM₁₀ | PI, 1.49 (-0.06–3.07) | per 10 µg/m³ | 1.01 (1.00–1.03) |
| Forastiere et al. (2008) | PM₁₀ | PI, 1.03 (0.28–1.79) | per 10 µg/m³ | 1.01 (1.00–1.02) |
| Aron et al. (2024) | PM₂.₅ | OR, 1.057 (1.00–1.116) | per 10 µg/m³ | 1.06 (1.00–1.12) |
| Bowe et al. (2019) | PM₂.₅ | HR, 1.36 (1.27–1.46) | per 10 µg/m³ | 1.36 (1.27–1.46) |
| Guo et al. (2024) | PM₂.₅ | HR, 1.036 (1.019–1.053) | per 1 µg/m³ | 1.42 (1.21–1.67) |
| Guo et al. (2024) | PM_1_ | HR, 1.032 (1.003–1.062) | per 1 µg/m³ | 1.37 (1.02–1.87) |
| Guo et al. (2024) | PM_1-2.5_ | HR, 1.085 (1.054–1.116) | per 1 µg/m³ | 2.26 (1.70–3.01) |
| Hu et al. (2025) | PM₂.₅ | HR, 1.06 (1.00, 1.14) | per 10 µg/m³ | 1.06 (1.00, 1.14) |
| Hu et al. (2025) | PM₂.₅ | HR, 1.10 (1.05, 1.16) | per 10 µg/m³ | 1.10 (1.05, 1.16) |
| Hu et al. (2025) | PM₁₀ | HR, 1.02 (0.99, 1.07) | per 10 µg/m³ | 1.02 (0.99, 1.07) |
| Hu et al. (2025) | PM₁₀ | HR, 1.05 (1.02, 1.08) | per 10 µg/m³ | 1.05 (1.02, 1.08) |
| Lim et al. (2018) | PM₂.₅ | HR, 1.19 (1.03, 1.39) | per 10 µg/m³ | 1.19 (1.03, 1.39) |
| Liu et al. (2020) | PM₂.₅ | RR, 1.60 (1.06-2.44) | per 10 µg/m³ | 1.60 (1.06-2.44) |
| Liu et al. (2020) | PM₂.₅ | RR, 1.22 (0.81-1.85) | per 10 µg/m³ | 1.22 (0.81-1.85) |
| Liu et al. (2020) | PM₂.₅ | RR, 1.59 (0.91-2.77) | per 10 µg/m³ | 1.59 (0.91-2.77) |
| Liu et al. (2020) | PM₂.₅ | RR, 1.13 (0.70-1.82) | per 10 µg/m³ | 1.13 (0.70-1.82) |
| Luo et al. (2023) | PM₂.₅ | HR, 0.98 (0.93, 1.04) | per 1.2 µg/m³ (IQR) | 0.85 (0.55–1.44) |
| Moon et al. (2024) | PM₂.₅ | HR, 1.03 (1.01, 1.06) | per 1 µg/m³ | 1.34 (1.10–1.79) |
| Oh et al. (2025) | PM₂.₅ | HR, 1.046 (1.007–1.086) | per 10 µg/m³ | 1.05 (1.01–1.09) |
| Paul et al. (2020) | PM₂.₅ | HR, 1.02 (0.999–1.042) | per 3.4 µg/m³ (IQR) | 1.10 (1.00–1.22) |
| Pinault et al. (2018) | PM₂.₅ | HR, 1.51 (1.39–1.65) | per 10 µg/m³ | 1.51 (1.39–1.65) |
| Pinault et al. (2018) | PM₂.₅ | HR, 1.52 (1.14–2.02) | per 10 µg/m³ | 1.52 (1.14–2.02) |
| Shan et al. (2020) | PM₁₀ | HR, 2.260 (1.732–2.950) | per 10 µg/m³ | 2.26 (1.73–2.95) |
| So et al. (2020) | PM₂.₅ | HR, 1.41 (1.05, 1.90) | per 4.39 µg/m³ (IQR) | 2.19 (1.12–4.32) |
| So et al. (2022) | PM₂.₅ | HR, 1.10 (1.04, 1.16) | per 5 µg/m³ | 1.21 (1.08–1.35) |
| Feng et al. (2024) | BC (PM2.5) | RR, 1.133 (0.894, 1.435) | per 2.06 μg/m³ (IQR) | 1.816 (1.21, 2.72) |
| Feng et al. (2024) | NH₄⁺ (PM2.5) | RR, 1.023 (0.904, 1.157) | per 7.13 μg/m³ (IQR) | 1.03 (0.89–1.19) |
| Feng et al. (2024) | NO₃⁻ (PM2.5) | RR, 1.012 (0.931, 1.100) | per 10.23 μg/m³ (IQR) | 1.01 (0.93–1.10) |
| Feng et al. (2024) | OM (PM2.5) | RR, 1.075 (0.989, 1.169) | per 12.33 μg/m³ (IQR) | 1.06 (0.97–1.16) |
| Feng et al. (2024) | SO₄²⁻ (PM2.5) | RR, 1.048 (0.932, 1.178) | per 7.53 μg/m³ (IQR) | 1.06 (0.92–1.24) |
| Wu et al. (2021) | PM₂.₅ | PI, 0.980 (0.377–1.583) | per 10 µg/m³ | 1.01 (1.00–1.02) |
| Wu et al. (2021) | PM₁₀ | PI, 0.877 (0.410–1.344) | per 10 µg/m³ | 1.01 (1.00–1.02) |
| Wu et al. (2022) | PM₂.₅ | HR, 1.49 (1.36, 1.64) | per 2.31 μg/m3 (IQR) | 2.29 (1.89–2.78) |
| Wu et al. (2022) | PM₁₀ | HR, 1.41 (1.29, 1.54) | per 3.25 μg/m3 (IQR) | 1.47 (1.27–1.7) |
| Wu et al. (2024) | PM₂.₅ | HR, 1.95 (1.86, 2.04) | per 10 µg/m³ | 1.95 (1.86, 2.04) |
| Wu et al. (2024) | PM_2.5-10_ | HR, 0.96 (0.90, 1.02) | per 10 µg/m³ | 0.96 (0.90, 1.02) |
| Yang et al. (2020) | PM₁₀ | RR, 1.0053 (1.0027, 1.0080) | per 10 µg/m³ | 1.00 (1.00–1.01) |
| Yin et al. (2023) | PM₂.₅ | OR, 1.0281 (1.0225, 1.0337) | per 3 33.8 µg/m³ (IQR) | 1.01 (1.00–1.02) |
| Yin et al. (2023) | PM_2.5-10_ | OR, 1.0192 (1.0132, 1.0252) | per 24.3 µg/m³ (IQR) | 1.01 (1.00–1.02) |
| Zhang et al. (2025) | PM₂.₅ | OR, 1.009 (1.005, 1.013) | per 10 µg/m³ | 1.01 (1.00–1.02) |
| Zheng et al. (2024) | PM₂.₅ | HR, 1.693 (1.422, 2.014) | per 2.249 µg/m³ (IQR) | 1.36 (1.26–1.46) |
| Zheng et al. (2024) | PM₁₀ | HR, 1.392 (1.175, 1.651) | per 3.163 µg/m³ (IQR) | 1.12 (1.05–1.20) |
| Gariazzo et al. (2023) | PM₂.₅ | %IR, 1.91 (-4.07–8.27) | per 10 µg/m³ | 1.00 (0.91–1.11) |
| Gariazzo et al. (2023) | PM₁₀ | %IR, 0.40 (-9.38–11.24) | per 10 µg/m³ | 1.02 (0.96–1.08) |
| Feng et al. (2021) | PM₂.₅ | HR, 1.25 (1.13–1.38) | per 10 μg/m³ | 1.25 (1.13–1.38) |
| Alessandrini et al. (2016) | PM₂.₅ | PC, 1.98 (0.54, 3.44) | per 10 µg/m³ | 1.02 (1.00–1.03) |
| Alessandrini et al. (2016) | PM₁₀ | PC, 2.43 (-0.39, 5.32) | per 14.4 µg/m³ | 1.02 (0.99–1.04) |
| Pope et al. (2014) | PM₂.₅ | HR, 1.13 (1.02–1.26) | per 10 µg/m³ | 1.13 (1.02–1.26) |
| Sui et al. (2020) | PM₂.₅ | ER, 1.03 (-0.28, 2.35) | per 10 µg/m³ | 1.01 (0.99–1.02) |
| Wu et al. (2025) | PM₂.₅ | IR, 3.41 (2.75, 4.08) | per IQR increase (2.45 µg/m³) | 1.15 (1.12–1.18) |
| Yu et al. (2025) | PM₂.₅ | HR, 0.93 (0.84-1.02) | per 10 µg/m³ | 0.93 (0.84–1.02) |
| Yu et al. (2025) | PM₁₀ | HR, 0.97 (0.92–1.03) | per 10 µg/m³ | 0.97 (0.92–1.03) |

**🞸 Some converted values presented here may differ slightly from those in the forest plots. This is due to the final pooling of these estimates using random-effects models in Stata software.**

**MPC, Mean percentage Change; HR, Hazzard ratio; PI, Percent increase; IR, increase risk; ER, Excess Risk;**

**Table S4.** Random and fixed effects meta-analyses for the associations between air pollutions and risk of diabetes-related mortality.

| **Air pollutant (Unit)** | **Diabetes-associated mortality** | | | | | | |
| --- | --- | --- | --- | --- | --- | --- | --- |
|  | **Number of datasets** | **REM Risk estimate (95% CI)** | **Heterogeneity** | | **FEM Risk estimate (95% CI)** | **Heterogeneity** | |
|  |  |  | ***I*^2^ (%)** | **Q** |  | ***I*^2^ (%)** | **Q** |
| PM_2.5_ (per 10 µg/m3 increment) | 41 | 1.123 (1.099 – 1.147) | 95.31 | 853.23 | 1.014 (1.010 – 1.017) | 95.31 | 853.23 |
| PM_10_ (per 10 µg/m3 increment) | 13 | 1.021 (1.007 – 1.035) | 81.68 | 65.50 | 1.007 (1.003 – 1.010) | 81.68 | 65.50 |
| Predicted PM_2.5_ | 1 | 1.070 (1.010 – 1.130) | - | - | 1.070 (1.010 – 1.130) | - | - |
| PM_2.5-10_ (per 10 µg/m3 increment) | 2 | 0.994 (0.948 – 1.040) | 61.47 | 2.60 | 1.009 (0.999 – 1.019) | 61.47 | 2.60 |
| PM_1_ (per 10 µg/m3 increment) | 1 | 1.370 (0.945 – 1.795) | - | - | 1.370 (0.945 – 1.795) | - | - |
| PM_1-2.5_ (per 10 µg/m3 increment) | 1 | 2.260 (1.605 – 2.915) | - | - | 2.260 (1.605 – 2.915) | - | - |

**Abbreviations: PM_2.5_**: Particulate Matter with a diameter of 2.5 micrometers or smaller; **PM_10_**: Particulate Matter with a diameter of 10 micrometers or smaller, **PM_1_**: Particulate Matter with a diameter of 1 micrometers or smaller, **PM_2.5-10_**: Particulate Matter with a diameter between 2.5 and 10 micrometers, **PM_1-2.5_**: Particulate Matter with a diameter between 1 and 2.5 micrometers, **REM**: Random effects meta-analyses, **FEM**, Fixed effects meta-analyses.

**Table S5.** meta regression between study variables and risk of diabetes-associated mortality due to PM_2.5_

| Study Variable** | Univariable Analyses | | | Multivariable Analyses | | |
| --- | --- | --- | --- | --- | --- | --- |
|  | **Coefficient^🞸^** | **95% CI** | ***P*-value** | **Coefficient^🞸^** | **95% CI** | ***P*-value** |
| Study quality (moderate) | -0.142 | -0.327 – 0.044 | 0.130 | -0.054 | -0.251 – 0.143 | 0.581 |
| Publication year | 0.002 | -0.013 – 0.017 | 0.773 | -0.005 | -0.032 – 0.021 | 0.693 |
| Implementation year- start | -0.004 | -0.013 – 0.005 | 0.361 | -0.003 | -0.017 – 0.012 | 0.695 |
| Exposure (long term) | 0.253 | 0.109 – 0.398 | 0.001 | 0.253 | 0.054 – 0.452 | 0.014 |
| Region (East Asia) | -0.051 | -0.224 – 0.121 | 0.548 | 0.074 | -0.163 – 0.312 | 0.529 |
| Region (Europe) | 0.077 | -0.164 – 0.319 | 0.521 | 0.102 | -0.158 – 0.363 | 0.430 |

🞸A positive coefficient indicates a larger hazard ratio (increased risk) associated with a unit increase in the covariate (e.g., a more recent publication year). A negative coefficient indicates a smaller hazard ratio (decreased risk).

****These variables omitted from the model because of collinearity:** Study quality: High / Exposure: Short term / Region: North America

I^2^ of model: 93.27%

**Adj R-squared = 20.06%** : This indicates that 20.06% of the between-study variance in effect sizes is explained by the variables included in the model**.**


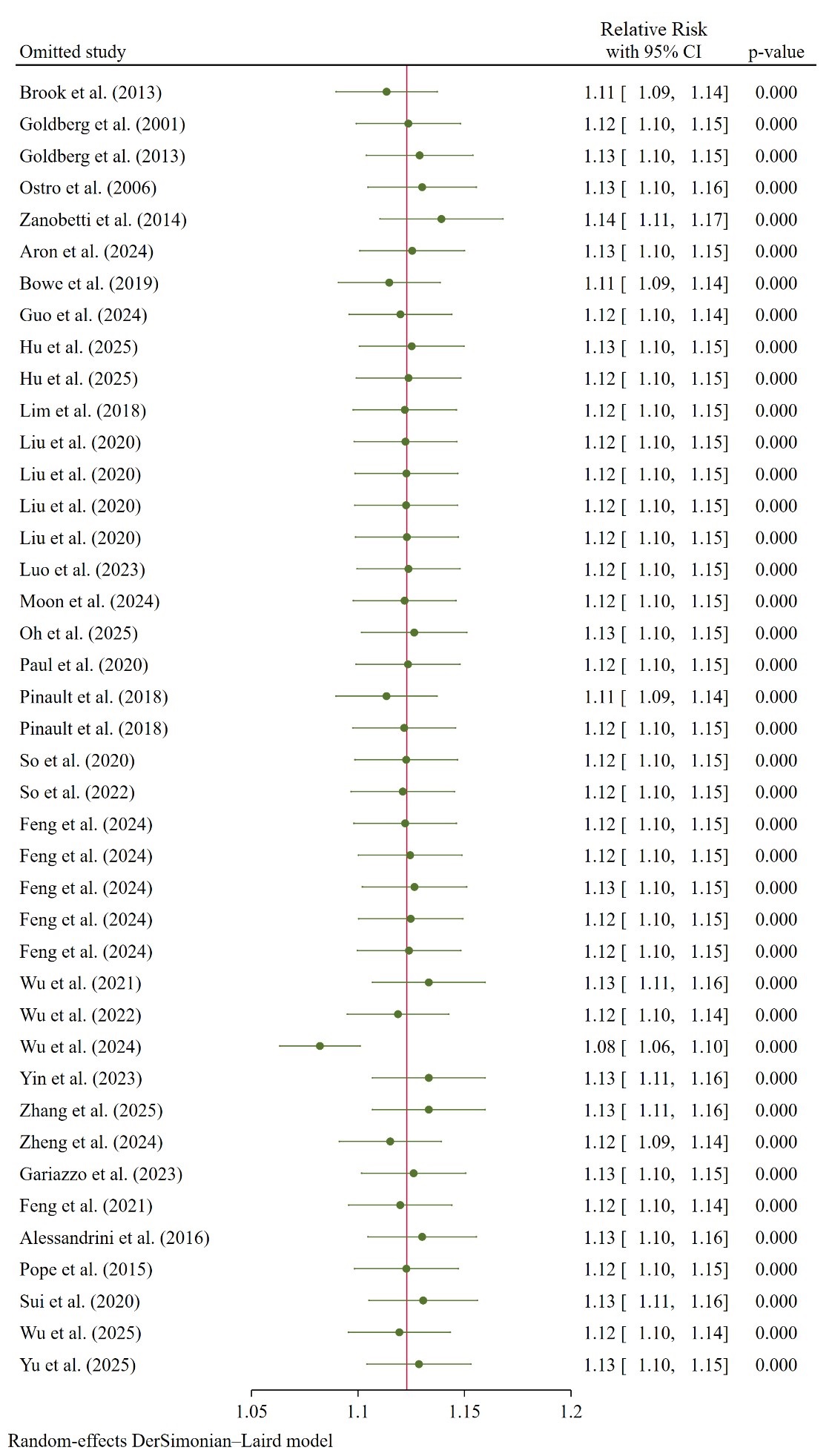


**Figure S1. Sensitivity analysis for the association between PM₂.₅ exposure and risk of diabetes-related mortality.**


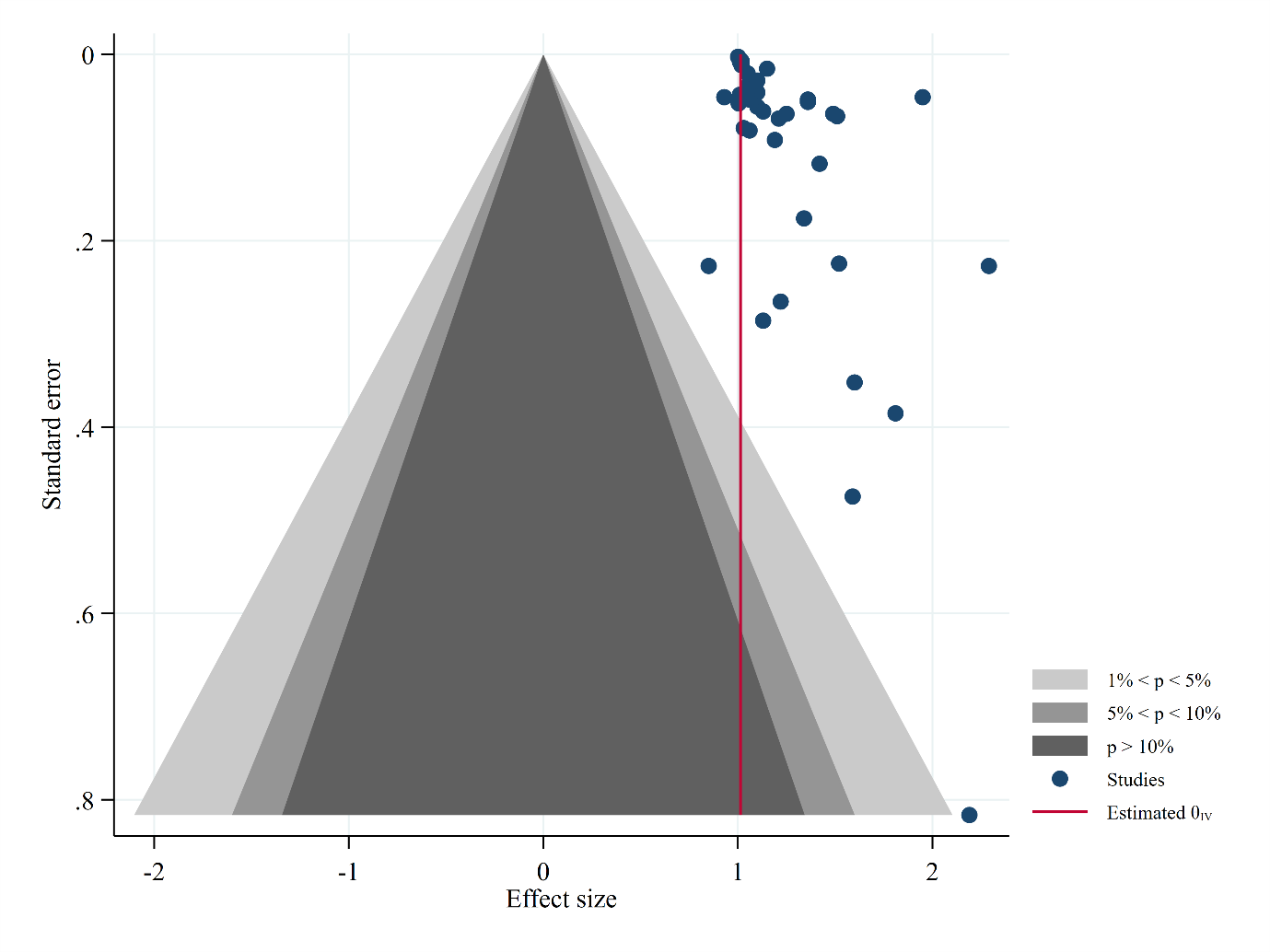


**Figure S2: Funnel plot for publication bias in PM_2.5_ and diabetes mortality studies.**


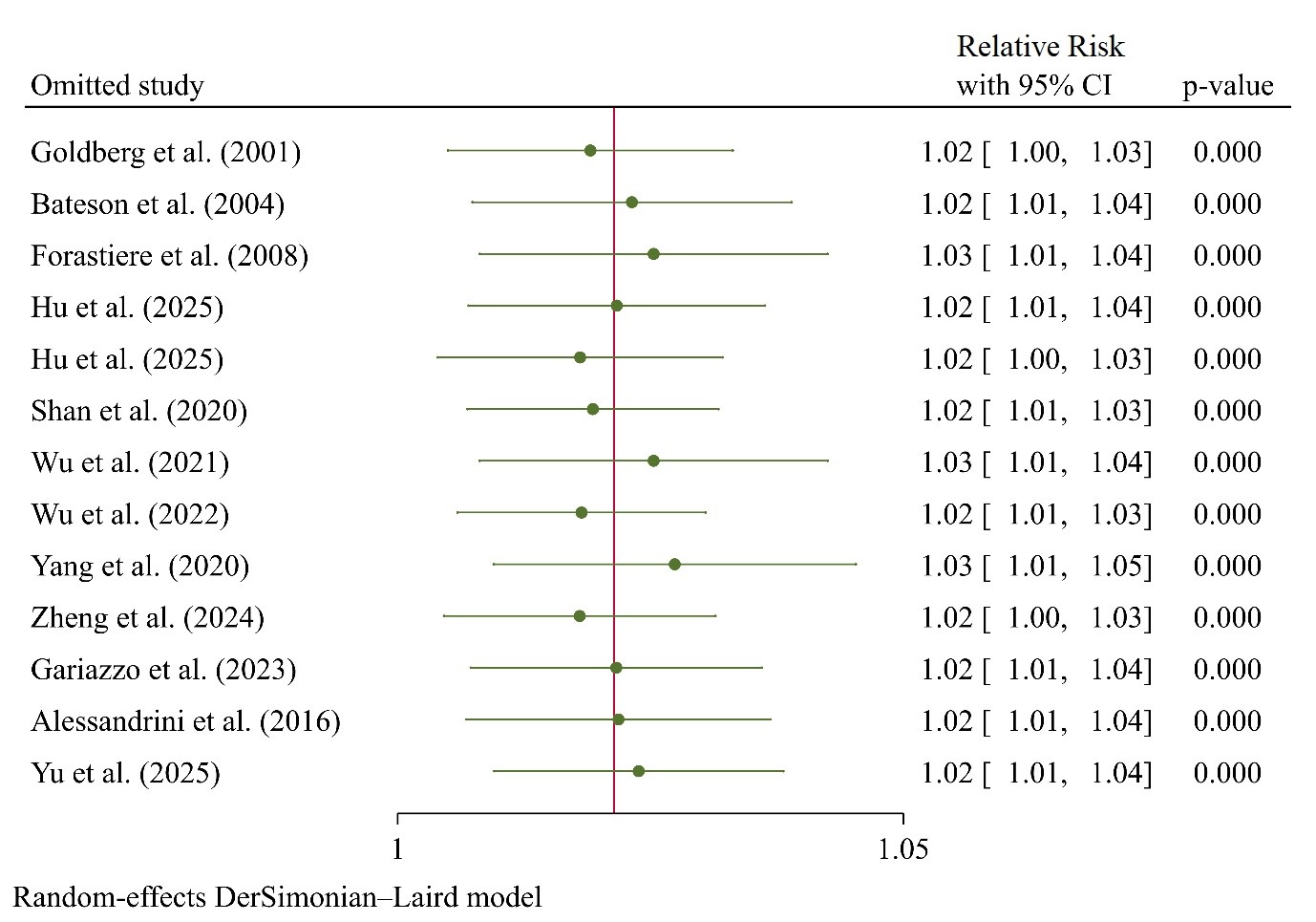


**Figure S3. Sensitivity analysis for the association between PM_10_ exposure and risk of diabetes-related mortality.**


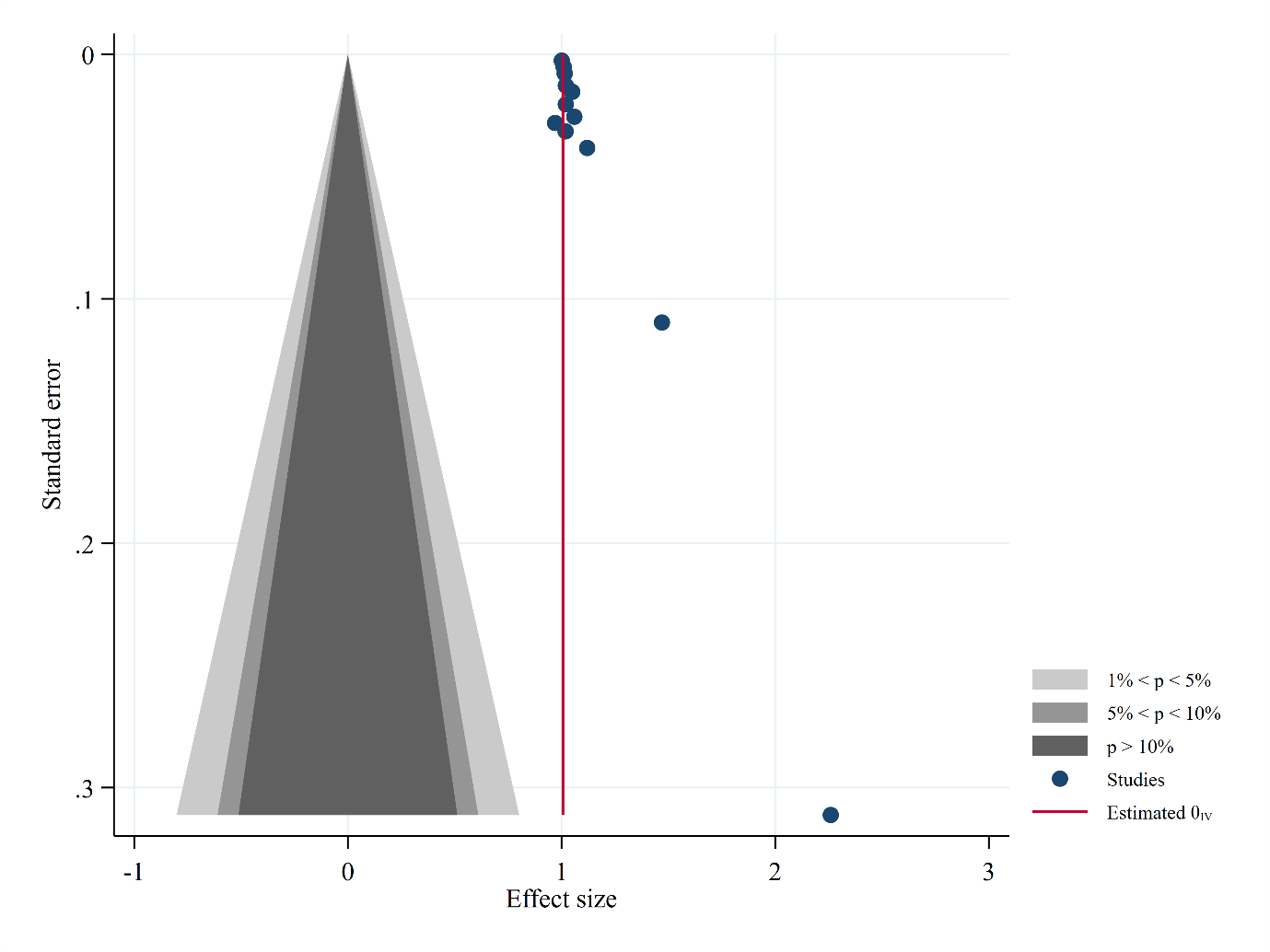


**Figure S4: Funnel plot for publication bias in PM10 and diabetes mortality studies.**
